# TMEM16A Inhibition Preserves Blood–Brain Barrier Integrity After Ischemic Stroke

**DOI:** 10.3389/fncel.2019.00360

**Published:** 2019-08-06

**Authors:** Pin-yi Liu, Zhi Zhang, Yi Liu, Xue-lian Tang, Shu Shu, Xin-yu Bao, Yan Zhang, Yue Gu, Yun Xu, Xiang Cao

**Affiliations:** ^1^Department of Neurology, Nanjing Drum Tower Hospital Clinical College of Nanjing Medical University, Nanjing, China; ^2^Department of Neurology, Nanjing Drum Tower Hospital, Medical School and The State Key Laboratory of Pharmaceutical Biotechnology, Institute of Brain Science, Nanjing University, Nanjing, China; ^3^Jiangsu Key Laboratory for Molecular Medicine, Nanjing University Medical School, Nanjing, China; ^4^Jiangsu Province Stroke Center for Diagnosis and Therapy, Nanjing, China; ^5^Nanjing Neuropsychiatry Clinic Medical Center, Nanjing, China

**Keywords:** blood–brain barrier, transmembrane protein 16A, intercellular adhesion molecule-1, ischemic stroke, NF-κB

## Abstract

The inflammatory response plays a pivotal role in Blood–Brain Barrier (BBB) destruction following ischemic brain injury. Enhanced leukocyte adhesion to vascular endothelial cells is an essential event in the inflammatory process. TMEM16A, a newly discovered protein regulating calcium-activated chloride channels, is widely expressed in eukaryotes. Recent studies have suggested that upregulated expression of TMEM16A is associated with the occurrence and development of many diseases. However, the role of TMEM16A in regulating BBB integrity after ischemic stroke has not been fully investigated. In this study, we found that TMEM16A is mainly expressed in brain endothelial cells and upregulated after ischemic stroke in the mouse brain. Caccinh-A01, an TMEM16A inhibitor that reduced its upregulation, attenuated brain infarct size and neurological deficits after ischemic stroke. ICAM-1 and MPO expression and BBB permeability were decreased after TMEM16A inhibitor administration. In addition, TMEM16A silencing rescued oxygen-glucose deprivation/reoxygenation (OGD/R)-induced transendothelial permeability *in vitro* accompanied by decreased ICAM-1 expression and leukocyte adhesion. Furthermore, our mechanistic study showed that TMEM16A knockdown alleviated NF-κB activation and nuclear translocation, indicating that TMEM16A knockdown downregulated OGD/R-induced ICAM-1 expression in an NF-κB-dependent manner. Finally, NF-κB inhibitor treatment also alleviated OGD/ R-induced BBB permeability, confirming that activated NF-κB and increased ICAM-1 are essential factors involved in ischemia-induced BBB damage. Thus, our research provides a promising treatment strategy against BBB destruction after ischemic stroke, and TMEM16A may become a potential target for the treatment of ischemic stroke.

## Introduction

The blood–brain barrier (BBB) is a barrier with highly selective permeability that protects the brain from many chemicals and bacterial threats. It is mainly composed of cerebral endothelial cells that are connected by Tight Junction (TJ) proteins (Obermeier et al., 2013). Clinically, BBB breakdown is a major contributing factor to neurological disorders including stroke and traumatic injury. Moreover, BBB breakdown is often associated with poor prognosis in ischemic stroke (Warach and Latour, 2004; Ji et al., 2017). Following ischemic stroke, BBB is disrupted, which allows leukocytes to infiltrate into the brain. Once leukocytes enter the brain tissue, they induce a secondary injury after reperfusion (Jiang et al., 2018). Intercellular adhesion molecule-1 (ICAM-1) plays an important role in leukocyte infiltration and it is mainly expressed on endothelial cells (Liu Y. et al., 2014; Cheng et al., 2018). Thus, inhibition of ICAM-1 after ischemic stroke prevents leukocyte adhesion and benefits BBB function recovery.

Transmembrane protein 16A (TMEM16A), also known as anoctamin-1, is a molecular identity of calcium-activated chloride channels expressed in a number of cell types and tissues including epithelial cells, sensory neurons, airway smooth muscle cells, cardiac muscle cells, and endothelial cells (Liu et al., 2010; Kunzelmann et al., 2012; Wu et al., 2014; Wang et al., 2018). TMEM16A has been demonstrated to play a critical role in mucus secretion, neural excitation, water-electrolyte balance and cell proliferation (Huang et al., 2012; Lin et al., 2015; Deng et al., 2016). Recently, it was found that TMEM16A participates in the pathophysiological processes during cardiac ischemia in cardiac vascular endothelial cells (Wu et al., 2014). Regarding stroke, TMEM16A is involved in hypertension-induced cerebrovascular remodeling through regulating smooth muscle cell proliferation (Wang et al., 2012). In human umbilical vein endothelial cells, TMEM16A was reported to bind Nox2 directly and then affect reactive oxygen species (ROS) generation (Ma et al., 2017). Before this study, it was unclear whether TMEM16A plays a role in brain endothelial cells and regulates BBB integrity after ischemic stroke.

In the present study, we found that TMEM16A is expressed in mouse brain endothelial cells and Human Brain Microvascular Endothelial Cells (HBMECs). Ischemia-induced BBB damage is accompanied by an increase in TMEM16A expression. Inhibition or knockdown of TMEM16A significantly protected against BBB disruption by downregulating ICAM-1 in a nuclear factor-kappa B (NF-κB)-dependent manner. Based on the above findings, our study identified a novel functional property of TMEM16A in brain endothelial cells, which may provide a new therapeutic target for the treatment of ischemia-induced BBB damage.

## Materials and Methods

### Reagents and Antibodies

T16ainh-A01, Caccinh-A01, 2,3,5-triphenyltetrazolium chloride (TTC), Evans Blue (EB) and FITC-dextran were purchased from Sigma-Aldrich (St. Louis, MO, United States). The antibodies against TMEM16A, CD31, Na/K ATPase, and mouse IgG were obtained from Abcam (Cambridge, MA, United States). Primary antibodies against NF-κB p65, phosphor-p65, and Myeloperoxidase (MPO) were from Cell Signaling Biotechnology (Hertfordshire, United Kingdom). Anti-GAPDH, anti-Lamin b, anti-HSP90, Dapi, and horseradish peroxidase (HRP)-linked secondary antibodies were obtained from Bioworld Biotechnology (Minneapolis, MN, United States). The antibodies targeting ZO-1, Occludin, Claudin-5, and ICAM-1 were provided by Invitrogen (Carlsbad, CA, United States). The DiI dye was purchased from Beyotime Biotech (Nantong, China). PDTC (a selective NF-κB inhibitor) was from Selleck Chemicals (LLC, Houston, TX, United States).

### Cell Culture

Primary microglia, astrocytes and neurons were cultured as we previously described (Cao et al., 2018; Jin et al., 2018). Briefly, primary microglia and astrocytes were prepared from 1–2 days old new born C57/BL6J mice. 10–12 days later, the microglia were separated from astrocytes by shaking the culture flasks at 180 rpm for 2 h. Primary cortical neurons were isolated from E15-17 C57/BL6J mice embryos. They were cultured and maintained in B27/neurobasal medium (Invitrogen) supplemented with glutamine for 10 days. Primary cultures of brain microvascular endothelial cells were prepared from cortices of adult mice. The cortices tissue was carved and digested with 0.2% collagenase/dispase containing 20 U/ml DNase I. Endothelial cells were grown in endothelial cell medium (ECM, ScienCell, Carlsbad, CA, United States) plus 5% fetal bovine serum (Biological Industries, Israel), antibiotics (100 units/ml penicillin and 100 μg/ml streptomycin) in a humidified atmosphere with 5% CO_2_ at 37°C. The purity of the microglia, astrocytes, neurons, and endothelial cells was greater than 90% as determined by Iba-1, GFAP, MAP-2, and CD31 staining.

HBMECs and THP-1 (human monocytic cell line) cells were purchased from ScienCell Research Laboratories. They were cultured in ECM and RPMI 1640 (HyClone, Logan, UT, United States). All experiments with HBMECs and THP-1 cells were used below 10 passages.

### Middle Cerebral Artery Occlusion Model in Mice

Two-month-old male C57/BL6J mice weighing about 25 g were purchased from the Animal Model Center of Nanjing University (Nanjing, Jiangsu, China). All procedures were approved by the Animal care and Use Committee at Nanjing University. In Middle Cerebral Artery Occlusion (MCAO) model, mice were anesthetized with pentobarbital sodium (45 mg/kg i.p.). A 6/0 sutures (Doccol Corporation, MA, United States) was inserted through internal carotid artery into the beginning of the middle cerebral artery (MCA) until the ipsilateral blood flow of MCA supply territory decreased to below 30% of baseline monitored by a laser Doppler flowmetry (Perimed Corporation, Stockholm, Sweden). After 60 min, the filament was withdrawn to allow blood reperfusion. Sham-operated groups underwent the same procedures expect for the insertion of the filament into the MCA. The mice were subjected to the dose of 5 mg/kg T16ainh-A01 and Caccinh-A01 or the same amount of saline by caudal vein injection within 15 min after the onset of reperfusion.

### Behavioral Test

For neurological function assessment, a Neurological severity scores (NSS) was used on day 1 and day 3 after MCAO. NSS is a composite test of motor, sensory, balance and reflex, which was graded on a scale of 0 to 18. Higher score means more serious symptoms. All tests were assessed in a blinded manner.

The rotarod test was used to assess motor deficit and sensorimotor coordination (Tatulli et al., 2018). The mice were trained for 3 days before MCAO using a rotarod device (RWD Life Science, Shenzhen, China). The rotating rod was accelerated from 4 to 40 rpm over 5 min. All the mice were trained three times a day, and each training lasts for 5 min with a 15 min interval for rest. On days 1 and 3 after MCAO, the time that each mouse fell from the rotating rod was recorded.

The grip strength test was used to measure the maximal muscular strength by mouse forelimbs (Akhtar et al., 2013). The mouse was suspended by the tail and approached to the T-bar of the apparatus (GS3, Bioseb, France). Once the mouse grasped the T-bar with two forelimbs, the animal was pulled backward until its grip is broken. The maximal force was recorded and six such tests were acquired per mouse. The mean force was calculated for analysis.

### Infarct Volume Calculation

Infarct volume was calculated as previously described (Meng et al., 2019). Mice were anesthetized and sacrificed 24 and 72 h after MCAO by 2% TTC (Sigma-Aldrich) staining. Mouse brains were sliced into five coronal sections (1-mm thick), and then stained with TTC at 37°C for 15 min. After 4% paraformaldehyde fixation, the slices are photographed and analyzed by ImageJ software. The value of infarct volume is calculated as a percentage of the whole brain after correcting for edema.

### Quantitation of BBB Permeability

Evans Blue extravasation is a classical method to examine the change of BBB permeability. Mice were injected with 2% EB (Sigma-Aldrich) after MCAO. Two hours later, animals were perfused with saline, and then the brains were cut into slices for analysis. Each hemisphere was weighed and homogenized in *N,N*-dimethyl formamide (Sigma-Aldrich). The samples were centrifuged, collected and quantified as previously described (Tang et al., 2018). Briefly, the supernatants were analyzed at 620 nm by a microplate reader (Tecan Trading AG, Switzerland). Results are expressed as micrograms of EB per gram of wet brain weight in comparison with a standard solution.

### Immunofluorescence Analysis

The mice were anesthetized and scarified via cardiac perfusion with pre-cold 0.9% saline followed by 4% paraformaldehyde. The brains were cut into 20 μm coronal sections by a cryostat microtome (Leica, Wetzlar, Germany). After blocking with 2% BSA, brain sections were immunostained with antibodies against CD31 (1:500), TMEM16A (1:200), ZO-1 (1:200), Occludin (1:200), Claudin-5 (1:500), and Mouse IgG (1:500) overnight at 4°C. The sections were washed with phosphate buffered saline (PBS) for three times and then incubated with the indicated secondary antibodies for 2 h in the dark at room temperature (RT). Thereafter, the cell nuclei were stained with DAPI (1:500). Images were photographed using a fluorescence microscope (Olympus BX51, Japan).

### Protein Preparation, Co-immunoprecipitation, and Western Blotting Analysis

Whole proteins were lysed with the lysis buffer (Thermo Fisher Scientific, Rockford, IL) for 30 min on ice. After centrifuging (12500 rpm) at 4°C for 15 min, the supernatants were immunoprecipitated with indicated antibodies overnight. The precleared Protein A/G Plus-agarose beads (Millipore, Billerica, MA, United States) were incubated with immunocomplexes for 4 h. The total protein concentrations were quantified by a BCA protein assay kit (Pierce Biotechnology, Rockford, IL, United States). Equal quantities of proteins were separated with 10–12% SDS-PAGE and then transferred onto PVDF membranes (Millipore). The membranes were blocked with 5% slim milk for 1 h at RT, followed by an overnight incubation with primary antibodies against TMEM16A (1:500), ZO-1 (1:500), Occludin (1:1000), Claudin-5 (1:500), MPO (1:1000), NF-κB p65 (1:1000), phosphor-NF-κB p65 (1:1000), Na/K ATPase (1:1000), HSP90 (1:1000), GAPDH (1:5000), and Lamin b (1:2000). Subsequently, the proteins were incubated with secondary antibody for another 2 h at RT and scanned with a Gel-Pro system (Tanon Technologies, Shanghai, China). The intensity of the blots was quantified with ImageJ software.

Cytoplasmic-and-nuclear or membrane-and-cytoplasmic protein fractions from HBMECs were extracted using NE-PER nuclear and cytoplasmic extraction assays or a Mem-PER Plus Membrane protein Extraction Kit (Thermo Fisher Scientific) as previously described (Weng et al., 2017). The following steps are the same as Western blotting.

### Real-Time PCR

Total RNA was extracted at 1, 3 and 7 days after MCAO with Trizol reagent (Invitrogen) and reversed transcribed to cDNA using a PrimeScript RT Reagent Kit (Takara, Dalian, China) according to the manufacturer’s instruction. Real-time PCR was performed on a StepOne Plus PCR System (Applied Biosystems, Foster City, CA, United States) with SYBER Green assay (Applied Biosystems). Corresponding primers were shown as follows: TMEM16A: F: GAAGCGGAAGCAGCGCTATG and R: AGTGGAGCCAGAGGGAAGGA; GAPDH: F: GCCAAGG CTGTGGGCAAGGT and R: TCTCCAGGCGGCACGTCAGA.

### Oxygen-Glucose Deprivation/Reoxygenation (OGD/R) Treatment

Human Brain Microvascular Endothelial Cells were treated with OGD to mimic acute ischemia *in vitro*. Briefly, HBMECs were seeded onto the inner surface of collagen-coated Transwell inserts (6.5 mm diameter, 0.4 μm pore size; Corning Costar, NY), which were placed in wells of a 24 well plate with complete growth media ECM. When the monolayer of HBMECs became confluent, culture mediums of the cells were replaced with glucose-free DMEM, and the cells were placed in a hypoxia chamber (Billups-Rothenberg, Del Mar, CA, United States) which was previously flushed with 95% N_2_/5% CO_2_. After 4 h incubation, the cells were returned to regular medium and incubated under normal conditions (95% O_2_/5% CO_2_, and 37°C) for another 20 h. Control cells were cultured in normal medium and environment all the time. For stimulation, HBMECs were preincubated with PDTC (100 μM) for 1 h prior to the OGD/R treatment.

### Measurement of Transendothelial Electrical Resistance (TEER)

The TEER of HBMEC monolayer was performed after 4 h OGD following by 20 h reoxygenation by using a portable epithelial voltohmmeter (EVOM, World Precision Instruments, Sarasota, FL, United States) according to the manufacturer’s instruction. All independent experiments were measured in triplicate. A Transwell without any cells was served as a blank control.

### FITC-Dextran Transendothelial Permeability Assay

The FITC-dextran transendothelial permeability assay was also applied to test the permeability of HBMEC monolayer. In brief, 0.1 mg/ml of FITC-labeled dextran (MW, 70000, Sigma-Aldrich) was added to the upper chamber after 4 h OGD following by 20 h reoxygenation. After incubation for another 20 min, 100 μl of supernatants from the lower compartment were analyzed by a microplate reader (excitation 490 nm, emission 520 nm). At least three independent experiments were performed.

### Transfection Experiments

The lentivirus mediated inhibition (Lv-shTMEM16A) or overexpression of TMEM16A (Lv-TMEM16A) and control lentivirus were obtained from GenePharma (Shanghai, China). HBMECs were cultured and incubated for 1 day with Lv-shTMEM16A or Lv-TMEM16A (MOI = 10), which were then replaced by fresh medium for another 3 days. The expression of TMEM16A was detected by Western blotting and immunofluorescence analysis.

### Assessment for THP-1 Cell Adhesion to HBMECs

To mimic leukocyte adhesion in the brain, THP-1 cells were used to adhere HBMECs *in vitro*. In brief, THP-1 cells were maintained in RPMI 1640 medium containing 10 μM DiI dye at 37°C for 20 min. After washing three times by PBS, the fluorescence-labeled THP-1 cells were added to the HBMEC monolayer (the ratio of HBMECs to THP-1 is 10:1) and allowed to adhere for another 30 min at 37°C. The mixed cells were then gently washed three times with PBS. Images were also photographed using Olympus BX51 fluorescence microscope and the fluorescence intensity was analyzed by Tecan microplate reader (excitation 549 nm, emission 565 nm). HBMEC monolayer was used as a blank control.

### Cell Viability Assays

Human Brain Microvascular Endothelial Cells were plated at 1 × 10^4^ cells/well in 96-well plates and treated with or without Lv-shTMEM16A. After OGD/R treatment for 24 h, the culture media were removed. HBMECs viability was detected by the Cell Counting Kit-8 (CCK-8; Dojindo Laboratories, Tokyo, Japan) according to the manufacturer’s instruction. The optical density was measured at 450 nm by a microplate reader.

### Statistical Analysis

Results are expressed as mean ± SEM or median and 25th and 75th percentiles for skewed variables. In addition, we performed Student’s *t*-test among 2 groups or one-way analysis of variance (ANOVA) followed by Bonferroni’s *post hoc* test among three or more groups for continuous variables with normal distribution, and Kruskal–Wallis test tests followed by Dunn–Bonferroni *post hoc* method for skewed variables. Statistical calculations were performed with SPSS 18.0. *P*-values < 0.05 were considered statistically significant.

## Results

### TMEM16A Is Mainly Expressed in the Brain Endothelial Cells and Upregulated After Ischemic Stroke

**FIGURE 1:**
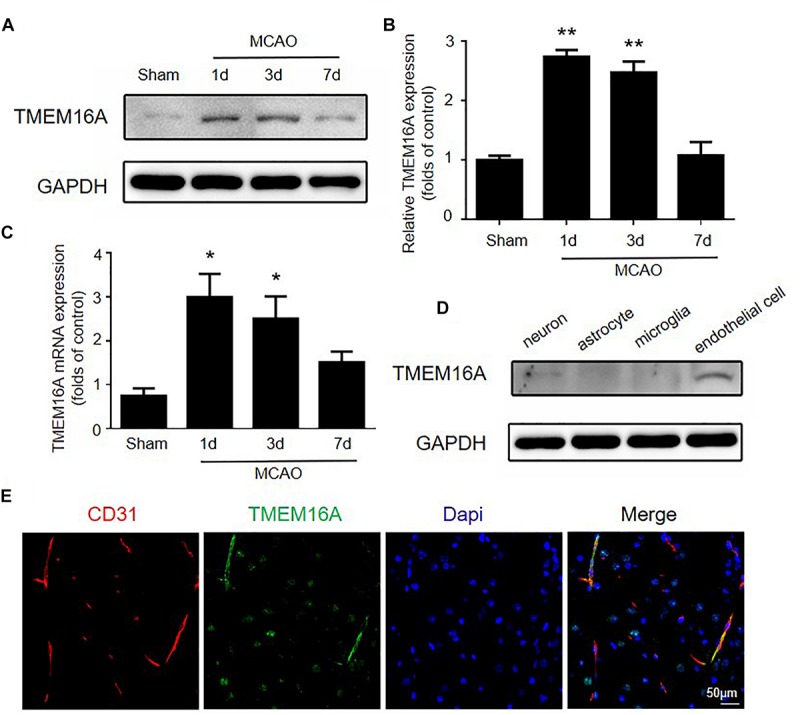
The expression of TMEM16A was upregulated after ischemic stroke. **(A,B)** TMEM16A protein and **(C)** mRNA expression 60 min following MCAO at different time points after the onset of reperfusion. **(D)** TEME16A protein expression in neurons, microglia, astrocytes, and brain endothelial cells. **(E)** Representative double-immunofluorescence staining of CD31 and TMEM16A in the mouse brain (bar = 50 μm). Values are the mean ± SEM for three mice in each group. One asterisk indicates *P* < 0.05 and two asterisks indicate *P* < 0.01 vs. sham-operated groups.

Several studies reported that TMEM16A is expressed in many endothelial cell types, including cardiac vascular endothelial cells, human umbilical vein endothelial cells, and mouse aortic endothelial cells (Suh et al., 1999; Zhong et al., 2000; Wu et al., 2014). However, little is known about the function and expression of TMEM16A in brain endothelial cells and the ischemia-induced inflammatory response. We measured TMEM16A expression after MCAO using Western blotting and real-time PCR assays. As shown in Figures 1A–C, TMEM16A protein and mRNA levels were markedly increased in the ipsilateral brain cortex on days 1 and 3 after MCAO. However, increased TMEM16A expression returned to basal levels on day 7 after MCAO. Furthermore, the expression profiles of TMEM16A were analyzed in four cell types in mouse brains. TMEM16A protein expression was more abundant in brain endothelial cells, than in neurons, microglia and astrocytes (Figures 1D,E). These findings suggest that TMEM16A may play an essential role in brain endothelial cells after ischemic stroke.

### Targeted Inhibition of TMEM16A Attenuated Brain Infarct Size and Neurological Deficits After Ischemic Stroke

**FIGURE 2:**
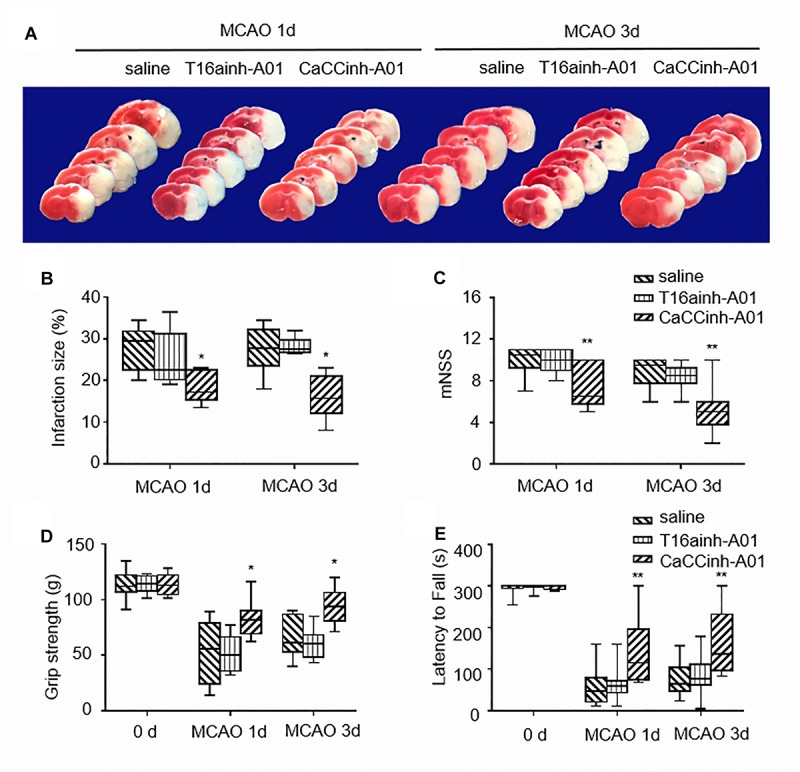
TMEM16A inhibitors reduced infarct volume and improved neurological outcomes after ischemic stroke. **(A)** Representative brain sections stained with TTC at different time points after MCAO. **(B)** Infarct volume (*n* = 6). **(C)** The results of NSS scores, **(D)** the grip strength and **(E)** the rotarod tests (*n* = 10). Values are the median (25th and 75th percentiles). One asterisk indicates *P* < 0.05 and two asterisks indicate *P* < 0.01 vs. MCAO-saline groups.

T16ainh-A01 and Caccinh-A01 are two selective inhibitors of TMEM16A. Both compounds have been demonstrated to inhibit TMEM16A activity, but Caccinh-A01 can also reduce TMEM16A protein levels by facilitating endoplasmic reticulum-associated, proteasomal turnover of TMEM16A (Bill et al., 2014). To determine the effect of TMEM16A inhibitors after ischemic stroke, the infarct size of mouse brains was measured by TTC at different time points. The results showed that Caccinh-A01, not T16ainh-A01, significantly reduced infarction: 29.50 (22.63–31.88) with MCAO-saline treatment vs. 17.25 (15.38–22.63) with MCAO-Caccinh-A01 treatment at 24 h (*p* < 0.05); 27.75 (23.63–32.25) with MCAO-saline treatment vs. 15.75 (12.13–21.23) with MCAO-Caccinh-A01 treatment at 72 h (*p* < 0.05; *n* = 6/group) (Figures 2A,B).

Neurological severity scores, the grip strength test and rotarod test were applied to test motor function, sensorimotor coordination,and muscular strength on each mouse after ischemic injury. As shown in Figures 2C–E, mice treated with Caccinh-A01 manifested better behavioral performance at 24 and 72 h than mice in the saline group (NSS: 10.50 (9.25–11.00) with MCAO-saline treatment vs. 6.50 (5.75–10.00) with MCAO-Caccinh-A01 treatment at 24 h, *p* < 0.01; 9.50 (7.75–10.00) with MCAO-saline treatment vs. 5.00 (3.75–6.00) with MCAO-Caccinh-A01 treatment at 72 h, *p* < 0.01. Grip strength test: 55.50 (24.00–79.25) with MCAO-saline treatment vs. 81.50 (69.25–90.00) with MCAO-Caccinh-A01 treatment at 24 h, *p* < 0.05; 61.50 (53.00–86.75) with MCAO-saline treatment vs. 94.00 (81.00–106.25) with MCAO-Caccinh-A01 treatment at 72 h, *p* < 0.05. Rotarod test: 47.50 (20.75–80.25) with MCAO-saline treatment vs. 115.00 (74.75–196.25) with MCAO-Caccinh-A01 treatment at 24 h, *p* < 0.01; 64.00 (46.50–104.00) with MCAO-saline treatment vs. 137.00 (96.75–232.50) with MCAO-Caccinh-A01 treatment at 72 h, *p* < 0.01. *n* = 10/group). Taken together, these data indicated that TMEM16A inhibition alleviated ischemic brain injury in MCAO mice.

### Targeted Inhibition of TMEM16A Attenuated-BBB Disruption After Ischemic Stroke

**FIGURE 3:**
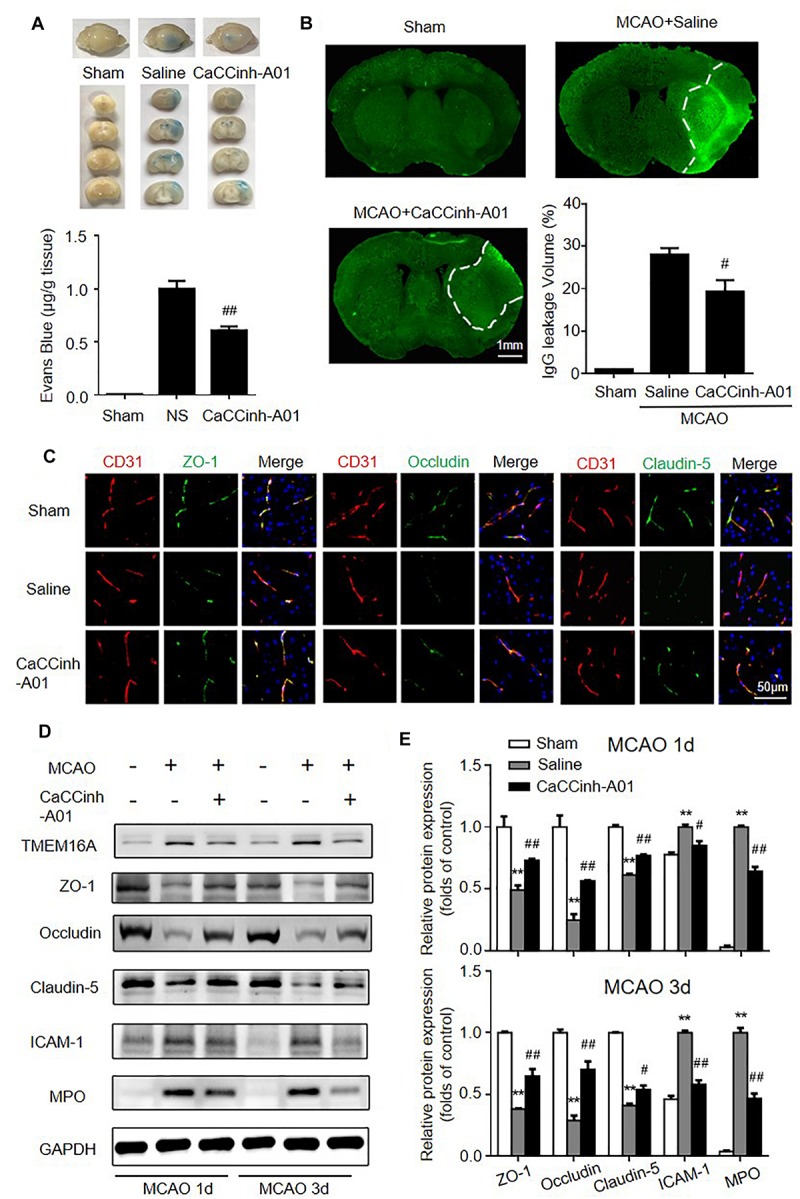
TMEM16A inhibition attenuated BBB disruption after ischemic stroke. **(A)** Evans blue staining and **(B)** IgG extravasation in sham-operated groups, MCAO-Saline groups and MCAO-Caccinh-A01 groups on day 3 after MCAO (bar = 1 mm). **(C)** Representative immunofluorescence staining of ZO-1, occludin and claudin-5 on day 3 after MCAO (bar = 50 μm). **(D,E)** Representative Western blotting results of TMEM16A, ZO-1, occludin, claudin-5, ICAM-1 and MPO on days 1 and 3 after MCAO. Values are the mean ± SEM. Two asterisks indicate *P* < 0.01 vs. sham-operated groups. One number sign indicates *P* < 0.05 and two number signs indicate *P* < 0.01 vs. MCAO-saline groups.

The effects of TMEM16A inhibition on BBB permeability were evaluated by Evans blue extravasation and IgG immunostaining. The results showed that increased Evans blue and IgG leakage were observed in the ischemic hemisphere on day 3 after MCAO, while there were significantly reduced Evans blue and IgG signals in Caccinh-A01 treated mice (Figures 3A,B). Since BBB is mainly composed of endothelial cells that are connected by TJ proteins, we further evaluated the protein expression of ZO-1, occludin, and claudin-5 by immunofluorescence and Western blotting. As shown in Figure 3C, ZO-1, occludin, and claudin-5 were dominantly and continuously distributed along the cerebral microvessels in a normal brain. However, the alignment of these three TJ proteins was seriously damaged in the ischemic hemisphere on days 1 and 3 after MCAO, indicating a disruption of BBB after ischemic stroke. The loss of ZO-1, occludin, and claudin-5 was attenuated in Caccinh-A01 treated mice compared with the MCAO-saline group. Consistent with this finding, Western blotting analysis confirmed that the reduction of these three TJ proteins was significantly reduced after Caccinh-A01 treatment (Figure 3D). At the same time, Caccinh-A01 markedly inhibited TMEM16A expression in the ischemic hemisphere after MCAO, while T16ainh-A01could not (Supplementary Figure S1).

Endothelial cells expressed a relatively low level of ICAM-1 in the homeostatic state. In hypoxic conditions, however, its expression was significantly increased. ICAM-1 upregulation facilitates neutrophil adhesion and infiltration, which are deleterious to BBB integrity. MPO was used to evaluate neutrophil accumulation. Thus, we also investigated the expression of ICAM-1 and MPO after MCAO with or without TMEM16A inhibition. We found that the protein levels of ICAM-1 and MPO were greatly downregulated in the MCAO-Caccinh-A01 group compared with the MCAO-saline groups (Figure 3D). These blots were quantified by densitometry (Figure 3E). Therefore, we concluded that TMEM16A inhibition could alleviate the disruption of BBB permeability after ischemic stroke by reducing ICAM-1 expression and neutrophil accumulation.

### TMEM16A Silencing Rescued OGD/R-Induced Transendothelial Permeability *in vitro*

**FIGURE 4:**
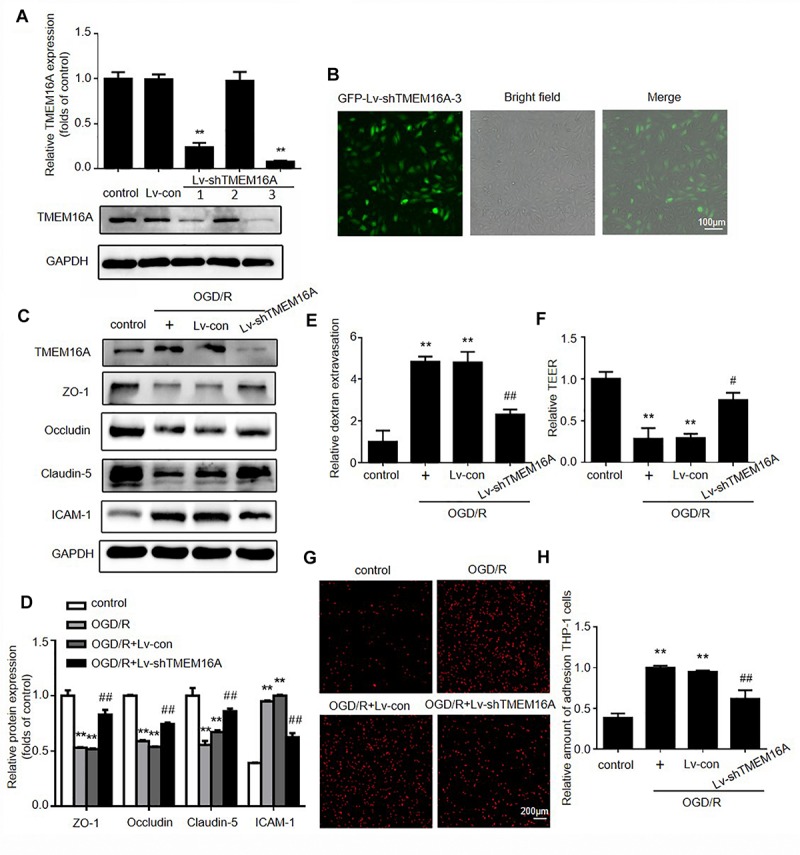
Silencing TMEM16A rescued OGD/R-induced transendothelial permeability *in vitro*. **(A)** TMEM16A expression was tested by Western blotting and **(B)** immunofluorescence after Lv-shTMEM16A infection for 3 days. **(C,D)** Representative Western blotting results of TMEM16A, ZO-1, occludin, claudin-5, and ICAM-1 after OGD/R treatments. **(E)** Quantification of transendothelial permeability detected by dextran leakage and **(F)** TEER assay. **(G,H)** Representative images of OGD/R-induced adhesion of THP-1 cells to HBMEC monolayers (bar = 200 μm). Values are the mean ± SEM. Two asterisks indicate *P* < 0.01 vs. control groups. One number sign indicates *P* < 0.05 and two number signs indicate *P* < 0.01 vs. OGD/R alone groups.

To further validate the effect of TMEM16A on the BBB, OGD/R models were used to mimic *in vivo* ischemia/reperfusion injury in an *in vitro* BBB model. OGD/R elevated TMEM16A expression in HBMECs, and we silenced TMEM16A expression with lentivirus before OGD/R. The transduction efficiency of Lv-shTMEM16A was tested by Western blotting and immunofluorescence (Figures 4A,B, *P* < 0.01). Both Lv-shTMEM16A-1 and Lv-shTMEM16A-3 were effective in silencing TMEM16A expression in HBMECs. Lv-shTMEM16A-3 was selected for the further experiments. After 4 h of OGD followed by 20 h of reoxygenation, the expression levels of TJ proteins (ZO-1, occludin and claudin-5) were significantly downregulated, accompanied by elevated TMEM16A. However, TMEM16A knockdown restored the protein levels of these three TJ proteins (Figures 4C,D). To further confirm this phenomenon, we isolated membrane protein to observe TJ proteins expression after OGD/R with or without Lv-shTMEM16A-3. The results also showed that TMEM16A knockdown upregulated the protein levels of ZO-1, occludin and claudin-5 after OGD/R in membrane fraction (Supplementary Figure S2A). In addition, ICAM-1 expression was upregulated in ODG/R, while TMEM16A silencing inhibited this upregulation (Figures 4C,D).

Furthermore, FITC-dextran permeability and TEER assays were applied to explore the role of TMEM16A in OGD/ R-induced transendothelial permeability. As expected, we found that TMEM16A knockdown significantly increased TEER values and decreased the OGD/R-induced FITC-dextran permeability (Figures 4E,F). To mimic leukocyte adhesion in the brain, human monocytic THP-1 cells were co-cultured with HBMECs. The results showed that THP-1 cells barely adhered to HBMECs in the untreated group. TMEM16A knockdown significantly reduced OGD/R-stimulated adhesion of THP-1 cells to HBMEC monolayers (Figures 4G,H). Therefore, these results demonstrated that TMEM16A knockdown also protected brain endothelial cells against OGD/R-induced transendothelial hyperpermeability, as well as THP-1 cell adhesion at the cellular level. Due to OGD/R may induce cell death, we also tested the cell viability after OGD/R with or without Lv-shTMEM16A. As shown in Supplementary Figure S2B, both OGD/R and TMEM16A knockdown did not significantly alter cell viability, which was consistent with our previously published article (Zhao et al., 2019). Taken together, these results indicated that the protective effect of TMEM16A knockdown on BBB integrity is independent of alterations in cell viability.

### TMEM16A Silencing Exerted a Protective Effect by Decreasing ICAM-1 via the NF-κB Signaling Pathway

**FIGURE 5:**
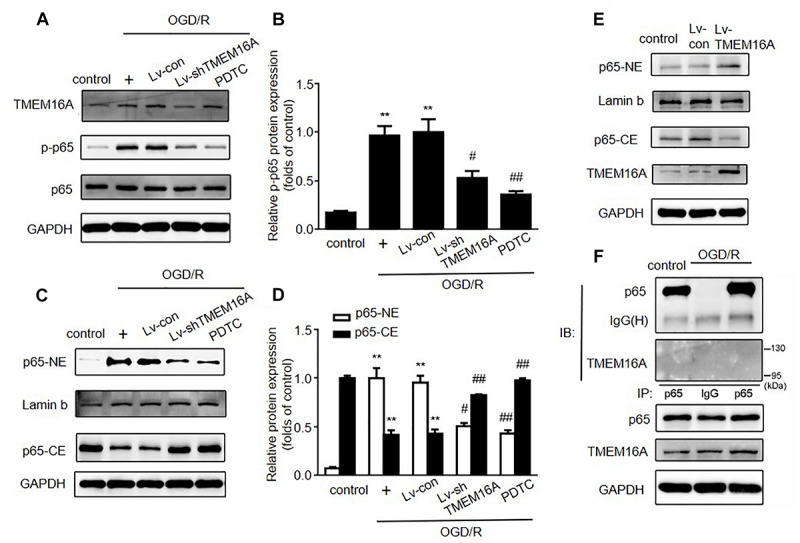
Silencing TMEM16A suppressed the activation of the NF-κB signaling pathway. **(A,B)** TMEM16A, NF-κB p-p65 and NF-κB p65 protein expression following OGD/R treatments. **(C–E)** NF-κB p65 protein expression in the cytoplasm and nucleus following OGD/R treatments in TMEM16A knockdown and overexpressing Human Brain Microvascular Endothelial Cells. **(F)** The interaction between TMEM16A and NF-κB p65 by co-immunoprecipitation. Values are the mean ± SEM. Two asterisks indicate *P* < 0.01 vs. control groups. One number sign indicates *P* < 0.05 and two number signs indicate *P* < 0.01 vs. OGD/R alone groups.

The expression of ICAM-1 can be regulated by the nuclear transcriptional factor NF-κB. To expand our understanding of the mechanisms underlying the protective effect of TMEM16A knockdown against BBB disruption, we next detected whether NF-κB signaling was involved in this process. As shown in Figures 5A,B, after 4 h of OGD followed by 20 h of reoxygenation, the expression of phosphor-NF-κB (p-p65) was significantly increased. After TMEM16A knockdown, the increase in p-p65 was prevented. Because activated NF-κB p65 translocates into the nucleus and then initiates the transcription of many inflammatory genes, such as ICAM-1. Western blotting was used to confirm whether TMEM16A silencing could also affect NF-κB p65 nuclear translocation. The results showed that a sharp increase in the nuclear translocation of NF-κB p65 was found after OGD/R treatments. However, TMEM16A knockdown prevented NF-κB p65 from entering the nucleus (Figures 5C,D). Furthermore, NF-κB p65 translocation was strongly enhanced after TMEM16A-overexpression in HBMECs (Figure 5E). To further determine the effect of TMEM16A on NF-κB p65, we analyzed the interaction between these two proteins. However, TMEM16A did not physically bind NF-κB p65 (Figure 5F). These data indicated that TMEM16A could regulate NF-κB p65 activity without direct physical interaction.

**FIGURE 6:**
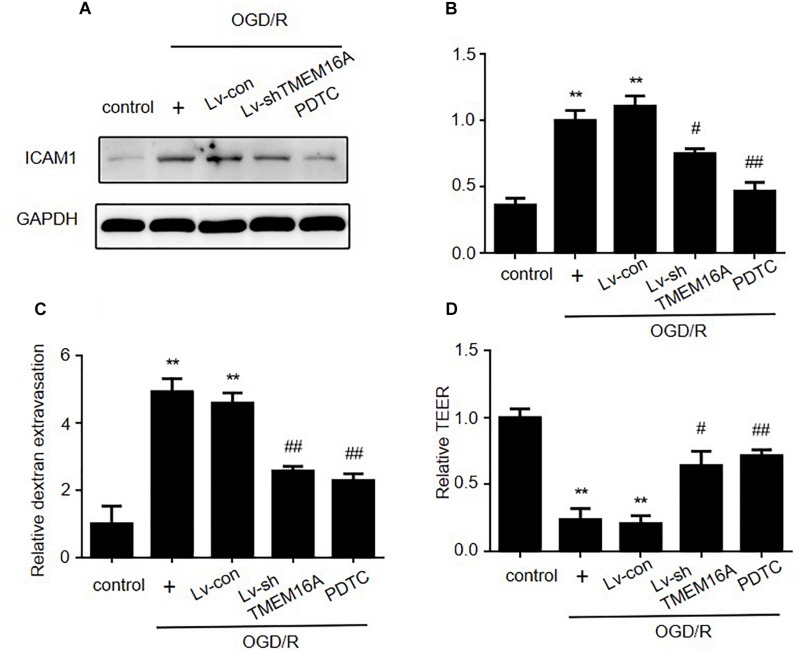
Silencing TMEM16A exerts its BBB protective effect by decreasing ICAM-1 expression via the NF-κB signaling pathway. **(A,B)** ICAM-1 protein expression following OGD/R treatments with TMEM16A knockdown, with or without PDTC treatment. **(C)** Quantification of transendothelial permeability detected by dextran leakage and **(D)** a TEER assay. Values are the mean ± SEM. Two asterisks indicate *P* < 0.01 vs. control groups. One number sign indicates *P* < 0.05 and two number signs indicate *P* < 0.01 vs. OGD/R alone groups.

PDTC, a selective inhibitor of NF-κB p65, was used to further determine the role of NF-κB signaling in ischemia-induced BBB disruption. Our present study showed that PDTC not only blocked NF-κB p65 translocation to the nucleus (Figure 5C) and reduced the expression of ICAM-1 (Figures 6A,B), but also increased TEER values and decreased the OGD/R-induced FITC-dextran permeability (Figures 6C,D). The protective effect of PDTC was equivalent to that of TMEM16A knockdown. Overall, TMEM16A silencing attenuates BBB disruption under hypoxic conditions by downregulating ICAM-1 levels via the NF-κB signaling pathway.

## Discussion

In the present study, we discovered that TMEM16A is upregulated in ischemia-induced brain injury *in vivo* and the OGD/R model *in vitro*, which is accompanied by ischemia-induced BBB damage. The TMEM16A inhibitor Caccinh-A01 attenuated brain infarct size, improved neurological outcomes and lowered BBB permeability after ischemic stroke. In addition, both the TMEM16A inhibitor and TMEM16A knockdown could also decrease neutrophil infiltration. We further revealed that the protective effects of TMEM16A inhibition or silencing were mediated by the downregulation of ICAM-1 in an NF-κB-dependent manner. A selective inhibitor of NF-κB (PDTC) was used to compare the effect of TMEM16A silencing. We found that similarly to TMEM16A knockdown, PDTC also reduced ICAM-1 expression and alleviated endothelial injury in the OGD/R model. Taken together, the results confirmed that TMEM16A silencing protected the BBB against ischemia or hypoxia partly through downregulating ICAM-1.

TMEM16A is a newly discovered molecular basis of calcium-activated chloride channels. It has 8 transmembrane segments and a highly conserved domain of unknown function (Ji et al., 2019). Zhang et al. (2013) found that TMEM16A is upregulated in an ovalbumin-stimulated chronic asthma mouse model, and functional blockage of TMEM16A prevents airway hyperresponsiveness. Following treatment with interleukin-13 (IL-13), Qin et al. (2016) demonstrated that both mRNA and protein levels of TMEM16A were increased in a human bronchial epithelial cell line. Treatment with TMEM16A inhibitor could suppress IL-13-induced TMEM16A-mediated mucus production. In addition, the mRNA and protein levels of TMEM16A were also significantly increased by exposure of cardiac vascular endothelial cells to hypoxia, indicating that TMEM16A participates in ischemia-induced damage in the heart (Wu et al., 2014). These studies revealed that TMEM16A plays a critical role in many diseases, while the effect of TMEM16A in ischemic stroke remains elusive. We first found that TMEM16A levels were markedly increased in the ipsilateral brain cortex on days 1 and 3 after MCAO and that TMEM16A is mainly expressed in brain endothelial cells. These results suggested that TMEM16A functioned mainly in endothelial cells during ischemia in the brain.

In the following experiments, we administered two selective inhibitors of TMEM16A (Caccinh-A01 and T16ainh-A01) to mice within 15 min after the onset of reperfusion. We did not find any articles that mentioned these two inhibitors in stroke research. However, several published articles related to other diseases have used T16ainh-A01 and Caccinh-A01. Pineda-Farias et al. (2015) intrathecally administered 10 μg/rat CaCCinh-A01 and T16ainh-A01 in a neuropathic pain model. Qi et al. (2018) prepared 0.12 mg/mouse T16ainh-A01 and injected it subcutaneously into sexually mature mice weighing 25–30 g. In our study, we used the same dose of T16ainh-A01 and CaCCinh-A01. The mice we used weighed approximately 25 g, and 5 mg/kg equals to 0.125 mg/mouse. As previously described, T16ainh-A01 and Caccinh-A01 have different inhibitory effects on TMEM16A. Caccinh-A01 was used to facilitate the degradation of TMEM16A in many types of tissues and T16ainh-A01 could only inhibit the channel activity of TMEM16A (Bill et al., 2014; Tian et al., 2018). In our present study, Caccinh-A01 obviously attenuated brain infarct size and neurological deficits by reducing the expression of TMEM16A. However, T16ainh-A01 could not reverse the upregulation of TMEM16A and did not affect the infarct size or the impaired sensorimotor functions, suggesting that ischemic-induced increase in TMEM16A exacerbated brain damage independent of TMEM16A biochemical activity.

The Blood–Brain Barrier is a highly selective semipermeable border that separates the central nervous system from the circulating blood. As a multicellular vascular structure, the BBB is composed of cerebral endothelial cells, pericytes, astrocyte end-feet, and basal membrane. Among them, endothelial cells, which have continuous intercellular TJ proteins, are the core component of the BBB. The disruption of the BBB exacerbates brain injury after ischemic stroke. Jiao et al. (2011) found that the expression levels of the TJ- associated proteins ZO-1, occludin and claudin-5 were significantly decreased after MCAO, which was consistent with our results. Our previous studies showed that reducing BBB leakage and damage could improve neurological outcomes, not only for MCAO-treated mice but also for acute ischemic stroke patients (Ji et al., 2017; Tang et al., 2018). In our study, we demonstrated that TMEM16A inhibition could reduce Evans blue staining and IgG extravasation and increase TJ proteins expression in brain tissues, leading to improved outcomes in an ischemic stroke mouse model. However, it is unclear how the TMEM16A inhibitor exerts its neuroprotective effects. ICAM-1 is a critical endothelia-associated transmembrane protein that stabilizes cell-cell interactions and facilitates leukocyte endothelial transmigration. Treatment with anti-ICAM-1 antibody reduced apoptosis in MCAO rats (Chopp et al., 1996). A number of studies have reported that MCAO significantly upregulated ICAM-1 expression and neutrophil infiltration at 24 h after the onset of reperfusion. Low-moderate ethanol consumption or metformin could alleviate ICAM-1 expression and neutrophil infiltration and improve BBB functions (Liu Y. et al., 2014; Xu et al., 2019). Therefore, inhibiting ICAM-1 upregulation in endothelial cells and accompanying neutrophil infiltration might be a promising strategy in treating ischemic stroke. In this study, the results showed that TMEM16A inhibition or knockdown could reduce ICAM-1 expression and neutrophil accumulation *in vivo* and *in vitro*.

Ischemic stroke and BBB disruption are age-related diseases/phenomena. A large contemporary cohort study by Fonarow et al. (2010) found that older stroke patients differ in clinical characteristics and experience higher in-hospital mortality than younger patients. Ritzel et al. (2018) also demonstrated that age alters the immunological response to stroke, as they found that neutrophil invasion into the brain was increased in aged animals and infiltrating monocytes produced higher levels of ROS and extracellular matrix-degrading enzymes (matrix metallopeptidase 9, MMP9), which, however, can be reversed by receiving bone marrow from young animals. Regarding age-related BBB change, a study using advanced MRI that measures BBB integrity showed that BBB dysfunction is an early event in aging brain (Montagne et al., 2015). Also, aged mice showed lower levels of occludin and ZO-1 than young mice (Elahy et al., 2015). In the present study, we used adult mice (about 2-month old) to perform MCAO, and aged mice could be used to confirm our results in the future.

It has been widely reported that NF-κB is a pivotal transcription factor with a role in the development of the inflammatory response (Yang et al., 2018; Zhang et al., 2018). In resting cells, NF-κB is located in the cytosol. In an activated state, however, NF-κB could be phosphorylated and translocated into the nucleus, promoting the expression of inflammation-associated genes, such as ICAM-1. The present study revealed that knockdown of TMEM16A suppressed NF-κB activation and nuclear translocation. However, overexpression of TMEM16A reversed this effect. These findings were consistent with the results of other studies in human bronchial epithelial cells and glioma cell lines (Liu J. et al., 2014; Lin et al., 2015). To further explore the association of TMEM16A and NF-κB, we also used immunoprecipitation assays to study their physical interaction. However, we failed to observe the direct binding of these two proteins, implying that the effect of TMEM16A on NF-κB might be related to another signaling protein. Tracey (2002) first discovered the neural mechanisms and functional anatomy of the cholinergic anti-inflammatory reflex. The α7 subunit of the nicotinic acetylcholine receptor (α7nAChR) is an important component in the vagus nerve-based cholinergic anti-inflammatory pathway, which inhibits NF-κB nuclear translocation. Using an α7nAChR agonist could inhibit proinflammatory cytokines. However, these effects were blocked by α7nAChR knockdown or α7nAChR antagonist (Wang et al., 2004; Parrish et al., 2008). Whether TMEM16A could affect α7nAChR expression needs to be investigated in the future. The selective inhibitor of NF-κB also alleviated the disruption of BBB permeability, confirming that NF-κB and ICAM-1 upregulation are essential factors involved in ischemia-induced BBB damage.

## Conclusion

In summary, our research demonstrated that inhibiting the aberrant upregulation of TMEM16A could reverse ischemia-induced BBB injury by downregulating ICAM-1 levels in an NF-κB signaling dependent manner. These findings suggested that TMEM16A may become a potential target for the treatment of ischemic stroke.

## Data Availability

The datasets generated for this study are available on request to the corresponding author.

## Ethics Statement

All procedures were approved by the Animal care and Use Committee at Nanjing University.

## Author Contributions

YX and XC conceived, designed, and coordinated the study. XC, ZZ, P-yL, YL, and X-lT performed the experiments and analyzed the data. X-yB, YZ, and YG helped with primary cells, culture, and induction of MCAO model. XC and P-yL wrote, revised, and checked the data analysis. All authors revised and approved the final version of the manuscript.

## Conflict of Interest Statement

The authors declare that the research was conducted in the absence of any commercial or financial relationships that could be construed as a potential conflict of interest.
