# Supplementary figures and images for: TMEM16A Inhibition Preserves Blood–Brain Barrier Integrity After Ischemic Stroke
Source: Front Cell Neurosci. 2019 Aug 6;13:360. doi: 10.3389/fncel.2019.00360 (PMC6691060; doi:10.3389/fncel.2019.00360)

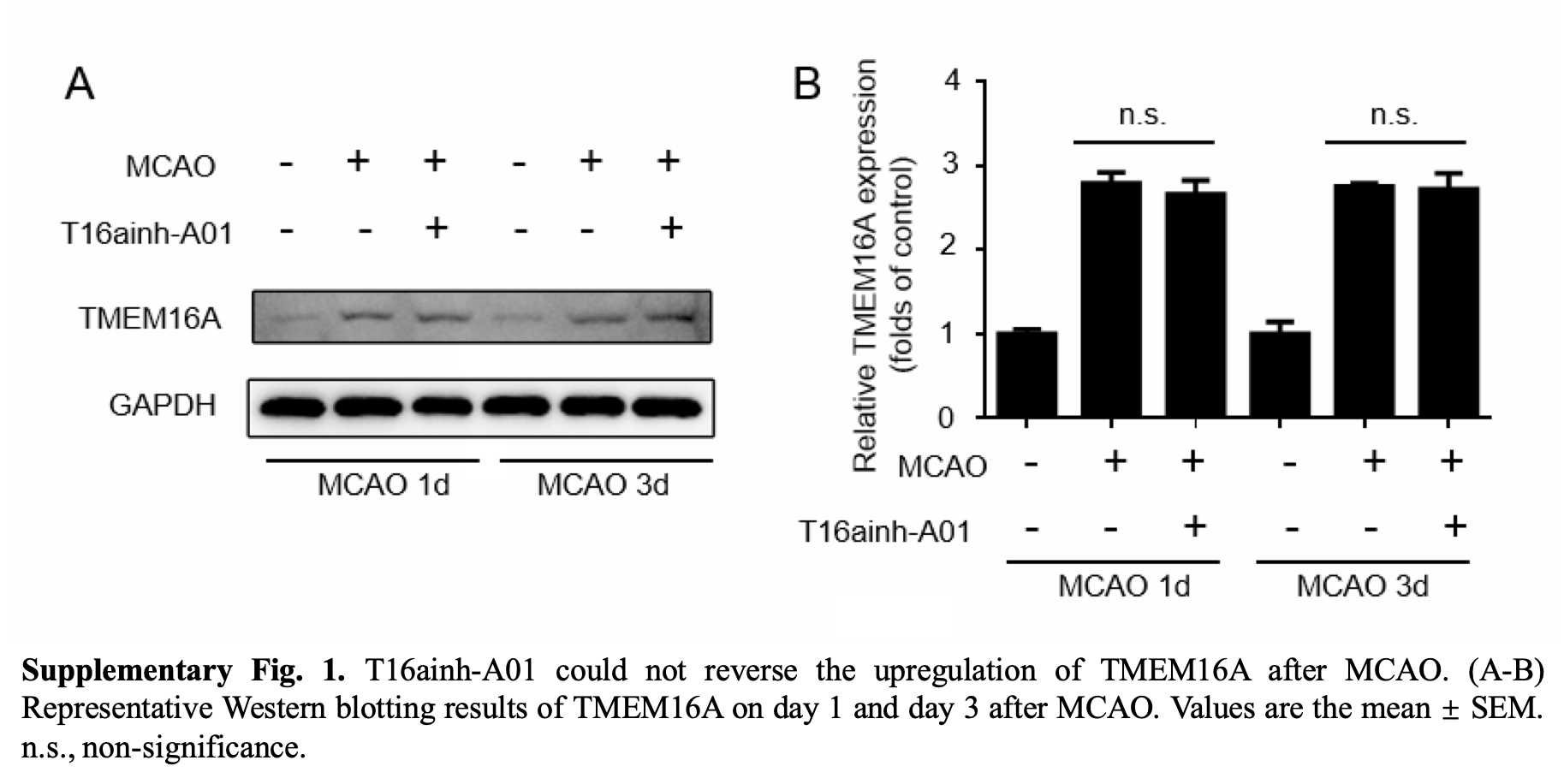

Supplement: Supplementary file 1 [file Image_1.TIF]

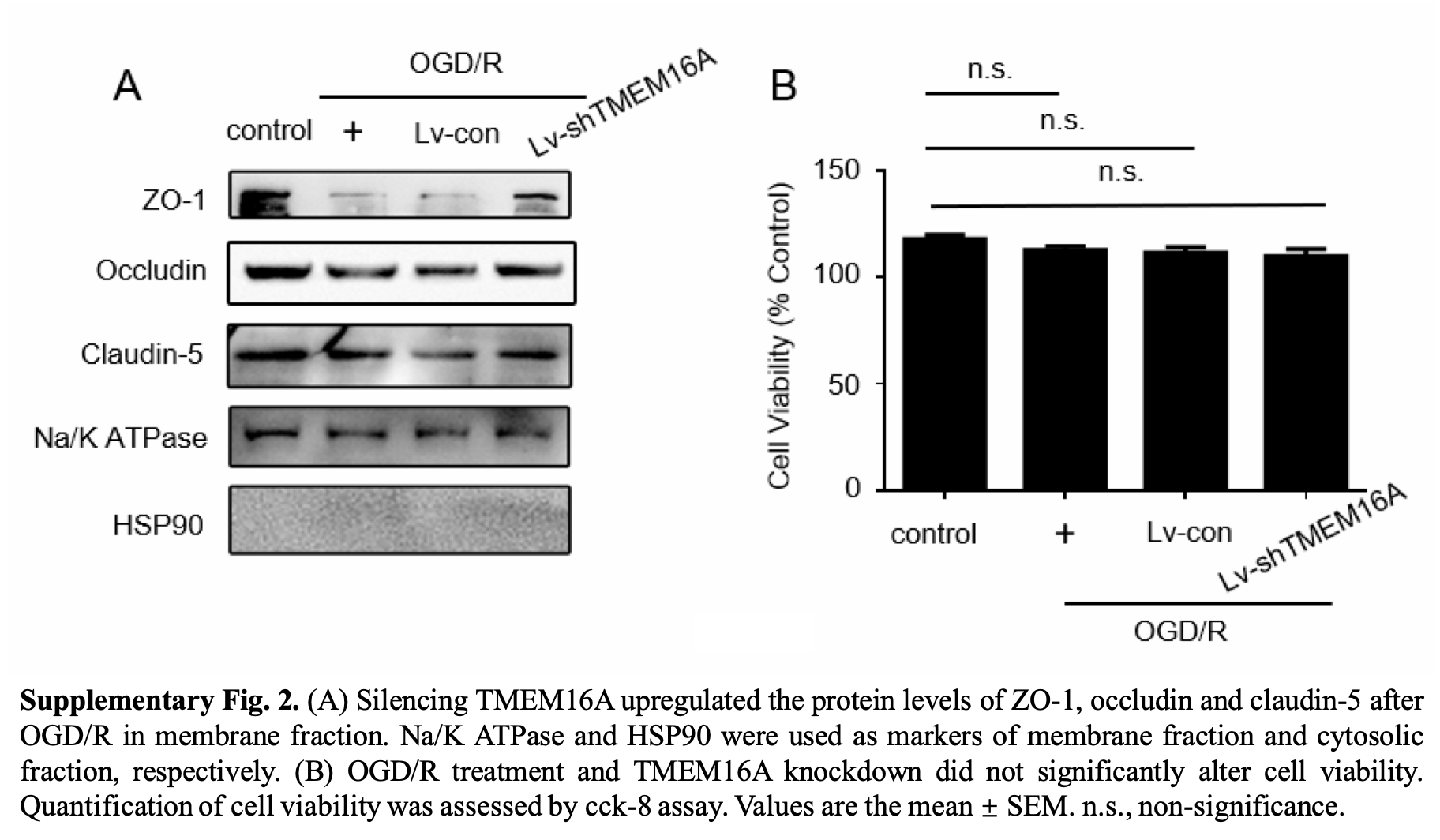

Supplement: Supplementary file 2 [file Image_2.TIF]
